# Insulin-Like Growth Factor Binding Protein (IGFBP-6) as a Novel Regulator of Inflammatory Response in Cystic Fibrosis Airway Cells

**DOI:** 10.3389/fmolb.2022.905468

**Published:** 2022-07-12

**Authors:** Onofrio Laselva, Maria Laura Criscione, Caterina Allegretta, Sante Di Gioia, Arcangelo Liso, Massimo Conese

**Affiliations:** ^1^ Department of Clinical and Experimental Medicine, University of Foggia, Foggia, Italy; ^2^ Department of Medical and Surgical Sciences, University of Foggia, Foggia, Italy

**Keywords:** cystic fibrosis, IGFBP-6, airway epithelial cells, dimethyl fumarate, TRIKAFTA, cytokine

## Abstract

Cystic Fibrosis (CF) patients are prone to contracting bacterial lung infections with opportunistic pathogens, especially *Pseudomonas aeruginosa*. Prolonged *P. aeruginosa* infections have been linked to chronic inflammation in the CF lung, whose hallmarks are increased levels of cytokines (i.e., TNF-α, IL-1β, IL-6) and neutrophil attraction by chemokines, like IL-8. Recently, insulin-like growth factor binding protein 6 (IGFBP-6) has been shown to play a putative role in the immune system and was found at higher levels in the sera and synovial tissue of rheumatoid arthritis patients. Moreover, it has been demonstrated that IGFBP-6 has chemoattractant properties towards cells of the innate (neutrophils, monocytes) and adaptive (T cells) immunity. However, it is not known whether IGFBP-6 expression is dysregulated in airway epithelial cells under infection/inflammatory conditions. Therefore, we first measured the basal IGFBP-6 mRNA and protein levels in bronchial epithelial cells lines (Wt and F508del-CFTR CFBE), finding they both are upregulated in F508del-CFTR CFBE cells. Interestingly, LPS and IL-1β+TNFα treatments increased the IGFBP-6 mRNA level, that was reduced after treatment with an anti-inflammatory (Dimethyl Fumarate) in CFBE cell line and in patient-derived nasal epithelial cultures. Lastly, we demonstrated that IGFBP-6 reduced the level of pro-inflammatory cytokines in both CFBE and primary nasal epithelial cells, without affecting rescued CFTR expression and function. The addition of a neutralizing antibody to IGFBP-6 increased pro-inflammatory cytokines expression under challenge with LPS. Together, these data suggest that IGFBP-6 may play a direct role in the CF-associated inflammation.

## Introduction

Cystic Fibrosis (CF) is one of the most common fatal genetic diseases affecting approximately 80,000 individuals worldwide. It is due to the absence or dysfunction of the CFTR protein resulting in the failure of chloride secretion and sodium hyperabsorption at the apical airway surface. This alteration leads to the dehydration of the airway surface, impaired mucociliary clearance and the accumulation of viscous mucus at the epithelial surface (Cuevas-Ocana et al., 2020). As a result, CF patients are prone to contracting bacterial lung infections with opportunistic pathogens, especially *Pseudomonas aeruginosa* (Lund-Palau et al., 2016). Prolonged *P. aeruginosa* infections have been linked to chronic inflammation in the CF lung, whose hallmarks are increased levels of cytokines (i.e., Tumor necrosis factor-alpha (TNF-α), Interleukin (IL)-1β, IL-6) and neutrophil attraction by chemokines, such as IL-8 (Reiniger et al., 2005). This inflammatory response is involved in worsening the damage to lung tissue, eventually leading to respiratory failure. The F508del-CFTR is the prototypical Class II CFTR mutation resulting in defective CFTR protein trafficking due to protein misfolding, reduced stability of the protein at the cell surface and dysfunctional channel gating. Following the clinical success of the first CFTR modulators, ivacaftor (VX-770) and lumacaftor (VX-809) (Guerra et al., 2020), the Food and Drug Administration (FDA) has recently approved a next generation CFTR modulator triple combination Tezacaftor (VX-661), Elexacaftor (VX-445) and Ivacaftor as a therapy (Trikafta™) for patients carrying at least one *F508del* allele (Middleton et al., 2019).

Insulin-like growth factors (IGFs) play a fundamental role in the regulation of cell metabolism and their activity is modulated by a family of six high-affinity IGF binding proteins (IGFBP1-6) (Hwa et al., 1999). Besides its preferential inhibition of IGF-II, IGFBP-6 has been shown more recently to exert IGF-independent effects and to play a putative role in the immune system (Bach, 2016; Liso et al., 2018). IGFBP-6 was found at higher levels in sera and synovial tissue of rheumatoid arthritis patients (Alunno et al., 2017). Interestingly, it has been demonstrated that IGFBP-6 has chemoattractant properties towards cells of the innate (neutrophils, monocytes) and adaptive (T cells) immunity (Conese et al., 2018). Moreover, monocyte-derived dendritic cells (Mo-DCs) showed higher expression of IGFBP-6 at hyperthermia (39°C) (Liso et al., 2017). Recently, it has been shown that IGFBP-6 can be found associated with extracellular vesicles (EVs), i.e., exosomes and microvesicles, when Mo-DCs are challenged with either hypertermia or an oxidative stress stimulus (Conese et al., 2020).

It has been demonstrated that IGFBP-6 is expressed in the epithelial layer of human bronchial organ cultures and in primary cultures of HBE cells (Sueoka et al., 2000) and correlates with the basal cell subpopulation marker *Krt14* (Plasschaert et al., 2018). Interestingly, it has been found to be differentially expressed in bronchial biopsies of asthmatic subjects (Vaillancourt et al., 2012). However, the precise role of IGFBP-6 in airway epithelial cells in pathophysiology, in particular in CF under infection/inflammatory conditions is not known.

Dimethyl fumarate (DMF) is an FDA approved anti-inflammatory drug approved for auto-immune or inflammatory diseases including psoriasis, neurodegenerative diseases and asthma (Seidel and Roth, 2013; Scuderi et al., 2020). Moreover, recently we demonstrated that DMF exhibited an anti-inflammatory and anti-oxidant effect on CF cells after different inflammatory stimulations (Laselva et al., 2021a).

In the current work, we compared the relative expression of IGFBP-6 in two different *in-vitro* testing models, the human bronchial epithelial cells line (CFBE41o-) and patient-derived nasal epithelial cultures (HNE). We found higher basal expression of IGFBP-6 cells bearing the *F508del* mutation compared to Wt-CFTR. Moreover, the IGFBP-6 expression was increased in both Wt- and F508del-CFTR CFBE and HNE cells under infection and inflammatory conditions. Interestingly, we found that IGFBP-6 reduced pro-inflammatory cytokines expression in a dose-dependent fashion while not altering Trikafta™-dependent F508del-CFTR functional expression.

## Materials and Methods

### Cell Culture

CFBE41o-cells stably expressing Wt- or F508del-CFTR were obtained from Dr. D. Gruenert (University of California, San Francisco, CA) and maintained in MEM (Biowest) supplemented with 10% FBS (Corning) and penicillin/streptomycin (Euroclone) at 37°C with 5% CO_2_ as previously described (Abbattiscianni et al., 2016). 0.5 μg/ml puromycin (Sigma-Aldrich) was used as a positive selection for Wt-CFTR and 2 μg/ml puromycin (Sigma-Aldrich) for the selection of F508del-CFTR CFBE41o-cells.

Primary nasal epithelial cells were obtained from CF patients enrolled in the Canadian Program for Individualised CF Therapy (CFIT) (https://lab.research.sickkids.ca/cfit; (Laselva et al., 2020b; Cao et al., 2020). Nasal epithelial cells were expanded and frozen as previously described (Hynds et al., 2019). The NIH-3T3 cells (Clone J2) were mitotically inactivated using 4 μg/ml mitomycin C (Merck) and used as feeder cells for NHE in combination with 10 µg Y-27632 (Selleck Chemicals). After expanded, HNE were seeded on collagen-coated transwell inserts (6.5 mm diameter, 0.4 µm pore size, Corning) as passage 3. Once confluent, the cells were differentiated for 18–20 days at an air-liquid interface (ALI) with basal differentiation media (PneumaCult-ALI, STEMCELL Technologies) (Laselva et al., 2020c).

The study was approved by the Research Ethics Board of The Hospital for Sick Children and St Michael’s Hospital (REB1000044783) (Toronto, ON, Canada). All study participants or their guardians signed an informed consent (Eckford et al., 2019; Laselva et al., 2020a).

### Ribonucleic Acid Extraction and Quantification Reverse Transcription Polymerase Chain Reaction

Cells were seeded at the density of 8 × 10^4^ cells/cm^2^ for 24 h in 6 wells plate. After 24 h, the cells were treated for 4 h with 1 μg/ml LPS derived from *P. aeruginosa* (Sigma-Aldrich) +/− a neutralizing antibody directed against IGFBP-6 (1 μg/ml, Abcam) (Liso et al., 2017), 30 ng/ml IL-1β (PeproTech) + 30 ng/ml TNFα (PeproTech) +/− 50 µM DMF (Sigma-Aldrich). For dose-response studies, the cells were treated with 1 μg/ml LPS + 0.2–200 ng/ml IGFBP-6. The cells were lysed and RNA was extracted according to the manufacturer’s protocol (Qiagen Mini Kit) as previously described (Laselva et al., 2020d). Total RNA was converted to cDNA with iSCRIPT cDNA synthesis kit (Biorad) and quantitative real-time PCR was performed using Ssfast EvaGreen (Biorad) and normalized to GADPH. Primer sets for IGFBP-6, TNFα, IL-6, IL-1β and GADPH have been reported previously (Liso et al., 2017; Laselva et al., 2021a).

### Immunoblotting

Samples were analysed by immunoblotting as described previously (Laselva et al., 2021b). Antibodies: mouse anti-CFTR clone 596 (UNC CFTR antibody distribution program by CFFT) at 1:1,000 dilution; rabbit anti-Calnexin (Sigma-Aldrich) at 1:10,000, rabbit recombinant anti-IGFBP-6 (Abcam) at 1:500 dilution were used. The blots were developed with Clarity Max Western ECL (Biorad) using ChemiDoc Imaging System (Biorad). Immunoblots were analyzed using the ImageJ software (NIH).

### IGFBP-6 Secretion

Cells were seeded at the density of 8 × 10^4^ cells/cm^2^ for 24 h in 6 wells plate. After 24 h, the cells were treated for 24 h with 1 μg/ml LPS derived from *P. aeruginosa* (Sigma-Aldrich), 30 ng/ml IL-1β (PeproTech) + 30 ng/ml TNFα (PeproTech) +/− 50 µM DMF (Sigma-Aldrich). Released IGFBP-6 in supernatants collected from cells was determined by ELISA kit (Thermo Fisher).

### CFTR Channel Function in CFBE Cells

CFBE cells were grown at 37°C for 5 days post-confluence submerged on 96-well clear bottom culture plates (Costar). Cells were treated for 24 h with 0.1% DMSO or 200 ng/ml IGFBP-6 (PeproTech) or 3 µM VX-661 + 3 µM VX-445 (Selleck Chemicals) +/− 200 ng/ml IGFBP-6. The cells were loaded with blue membrane potential dye (Molecular Devices) and dissolved in a chloride-free buffer for 30 min at 37°C. The plate was then read in a fluorescence plate reader (FilterMax F5, Molecular Devices) at 37°C (Laselva et al., 2019). After reading the baseline fluorescence for 5 min, CFTR was stimulated using 1 µM forskolin (FSK) for Wt-CFTR or 10 µM FSK + 1 µM VX-770. CFTR-mediated depolarization of the plasma membrane was detected as an increased in fluorescence. Then, CFTR inhibitor (CFTRinh-172, 10 µM) was added to deactivate CFTR. In our analyses, the peak changes in fluorescence to CFTR agonists were normalized relative to fluorescence immediately before agonist (forskolin ± VX-770) addition. The max activation expressed as % was calculating by difference between the peak of maximal CFTR activation and the last point of baseline (Laselva et al., 2022).

### Statistical Analysis

All the data are represented as mean ±  SEM of at least three replicates. GraphPad 8.0 software was used for all statistical analysis. The paired two-tailed *t*-test or one-way ANOVA were conducted as appropriate with a significance level of *p* < 0.05. Data with multiple comparison were assessed using Turkey’s multiple comparison test with *α* = 0.05.

## Results

### IGFBP-6 Expression Is Higher in F508del-CFTR CFBE Cells and Is Further Increased by LPS-Stimulation

**FIGURE 1:**
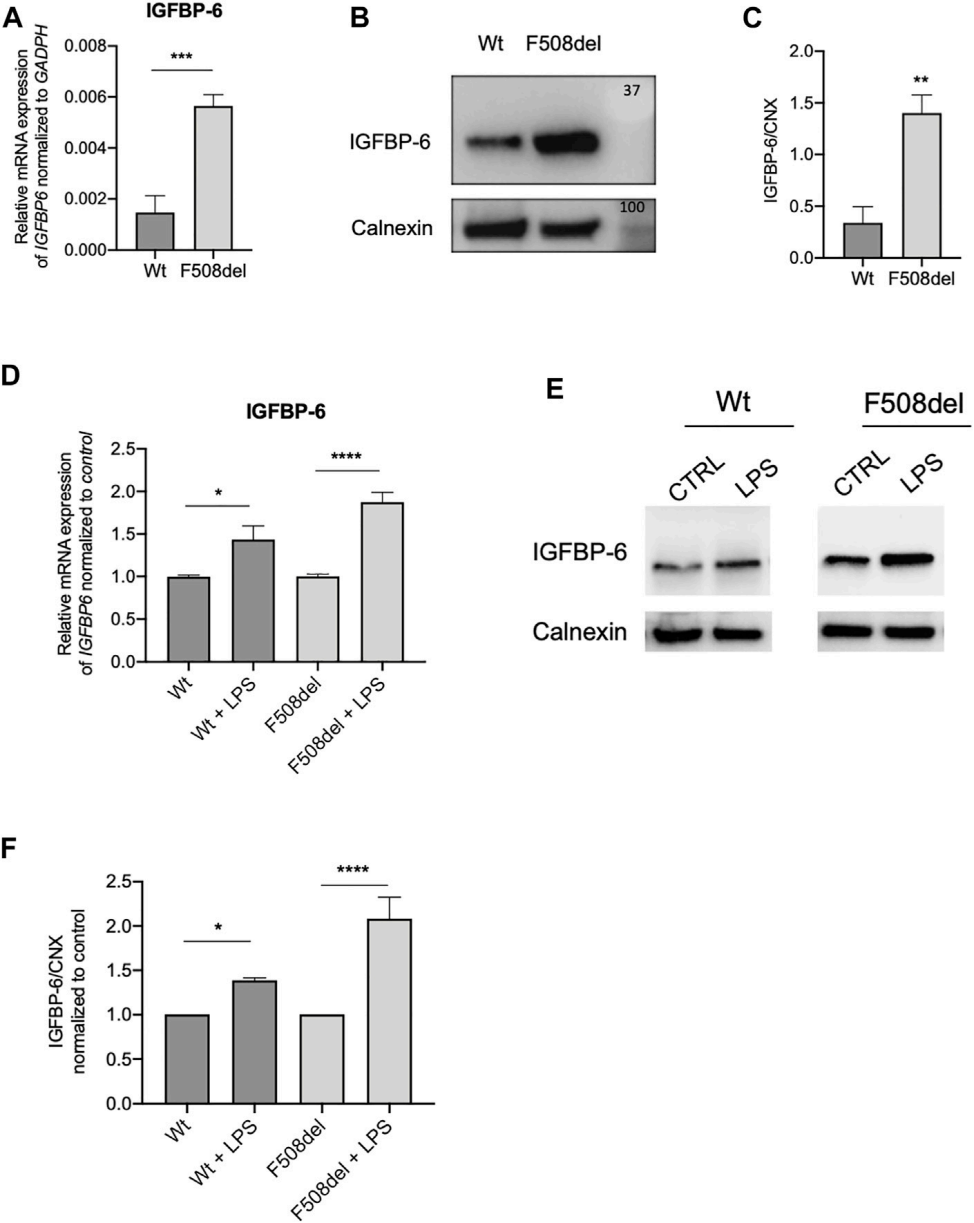
IGFBP-6 shows higher expression in F508del-CFTR CFBE cells and is further increased after LPS treatment. **(A)** Total RNA was extracted and qRT-PCR was performed in order to quantify IGFBP-6 mRNA normalized to GADPH as housekeeping gene. Data represent the mean ± SEM (*n* = 3). Statistical significance tested using paired two-tailed *t*-test. **(B)** Immunoblots of basal expression of IGFBP-6 in Wt and F508del-CFTR CFBE cells. **(C)** Data represent the mean ± SEM of the ratio IGFBP-6/Calnexin (*n* = 3). Statistical significance tested using paired two-tailed *t*-test. **(D,E)** Cells were treated with 1 μg/ml LPS for 4 h. Total RNA was extracted and qRT-PCR was performed in order to quantify IGFBP-6 mRNAs normalized first to GADPH as housekeeping gene and then to control untreated cells. Data represent the mean ± SEM (*n* = 4). Statistical significance tested using two-way ANOVA with Tukey’s multiple comparisons test **(F)** Immunoblots of IGFBP-6 following treatment with 1 μg/ml LPS for 24 h. **(G)** Data represent the mean ± SEM of the ratio IGFBP-6/Calnexin normalized to Calnexin control (*n* = 3–4). *p < 0.05; **p < 0.01; ****p < 0.0001.

Since previous studies demonstrated that IGFBP-6 is expressed in human bronchial epithelial cells (Sueoka et al., 2000), we employed qRT-PCR to determine the IGFBP-6 expression in non-CF (Wt-CFTR CFBE) and CF (F508del-CFTR CFBE) cells. Interestingly, at basal level, the mRNA IGFBP-6 expression in F508del-CFTR CFBE cells is higher than in Wt-CFTR CFBE cells (Figure 1A). To validate the mRNA expression studies at the protein level, IGFBP-6 was also studied by immunoblotting studies. According to qRT-PCR data, immunoblotting revealed that IGFBP-6 expression is higher in F508del-CFTR CFBE cells (Figures 1B,C).

Since CF airways are characterized by *P. aeruginosa* chronic infection, we treated the cells with LPS from *P. aeruginosa* as a surrogate for whole bacteria. As shown in Figures 1D,F,G and Supplementary Figure S1A, LPS increased the expression of IGFBP-6 in both Wt and F508del-CFTR CFBE cells.

### Dimethyl Fumarate Reduces the IGFBP-6 Level Under Infection/Inflammatory Conditions

**FIGURE 2:**
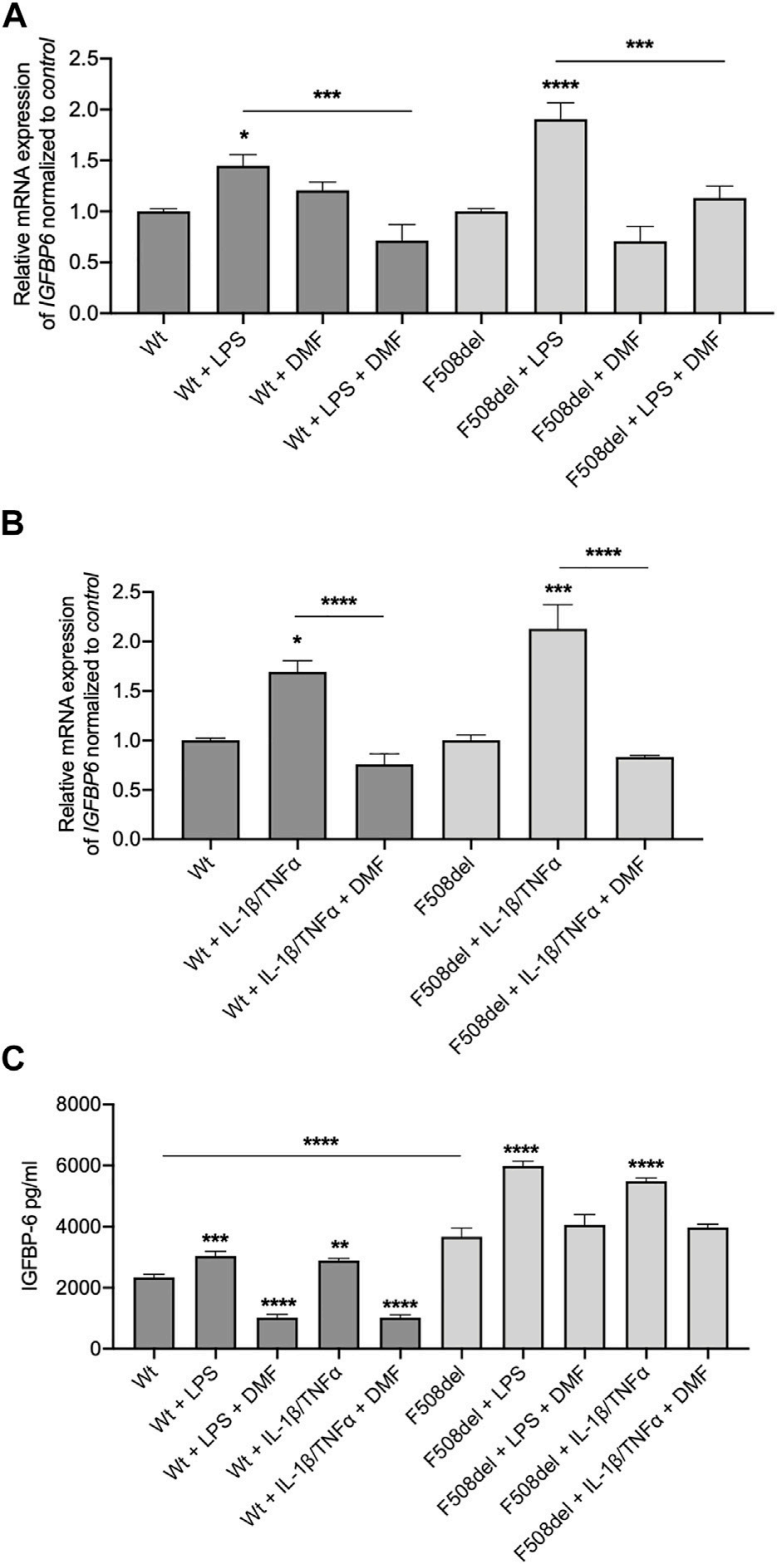
IGFBP-6 is reduced by Dimethyl fumarate. **(A)** Cells were treated with 0.1% DMSO, 1 μg/ml LPS ± 50 µM DMF or **(B)** PBS, 30 ng/ml IL-1β + 30 ng/ml TNFα ± 50 µM DMF for 4 h. Total RNA was extracted and qRT-PCR was performed in order to quantify IGFBP mRNA normalized to GADPH as housekeeping gene and then to control untreated cells. Data represent the mean ± SEM (*n* = 4). **(C)** IGFBP-6 secretion after stimulation with 0.1% DMSO, 1 μg/ml LPS, 30 ng/ml IL-1β + 30 ng/ml TNFα ± 50 µM DMF for 24 h. Data represent the mean ± SEM (*n* = 3). *p < 0.05; **p < 0.01; ***p < 0.001; ****p < 0.0001. Statistical significance tested using two-way ANOVA with Tukey’s multiple comparisons test to the control (Wt or F508del) for each cell line.

Recently we demonstrated that DMF reduced the inflammatory response in a bronchial epithelial cell line (Laselva et al., 2021a). Interestingly, while DMF did not reduce basal IGFBP-6 mRNA expression in both CFBE cell lines, it significantly reduced the IGFBP-6 expression when the cells were co-treated with DMF and LPS (Figure 2A and Supplementary Figure S1A).

To better understand the pathophysiology role of IGFBP-6 protein in CF, we measured IGFBP-6 expression in CFBE cells under inflammatory condition. Therefore, we treated the cells with IL-1β and TNFα to mimic the inflammatory milieu of CF airways. As shown in Figure 2B and Supplementary Figure S1A, the IGFBP-6 expression was increased in both Wt- and F508del-CFTR CFBE cells. Interestingly, DMF reduced to the basal level the IGFBP-6 expression in both CFBE cell lines in the presence of inflammatory cytokines.

Interestingly, also IL-8 mRNA levels followed the same behaviour of IGFBP-6, that is they were significantly increased upon challenge with either LPS or IL1β/TNF-α stimuli and were significantly reduced by DMF (Supplementary Figure S1B).

To validate the mRNA expression studies, we measured the IGFBP-6 protein level in the conditioned medium by ELISA. Interestingly, the IGFBP-6 levels were significantly higher in F508del-CFTR CFBE cells than in Wt-CFTR CFBE (Figure 2C). Moreover, the IGFBP-6 protein levels increased in both cell lines after LPS and IL-1β/TNFα treatment and were reduced in the presence of DMF (Figure 2C). Overall, these results suggest that IGFBP-6 plays a role in the inflammatory response operated by airway epithelial cells and that DMF acts as an agent blocking IGFBP-6 upregulation.

### IGFBP-6 Downregulates Pro-inflammatory Cytokine Expression Without Affecting CFTR Expression and Function

**FIGURE 3:**
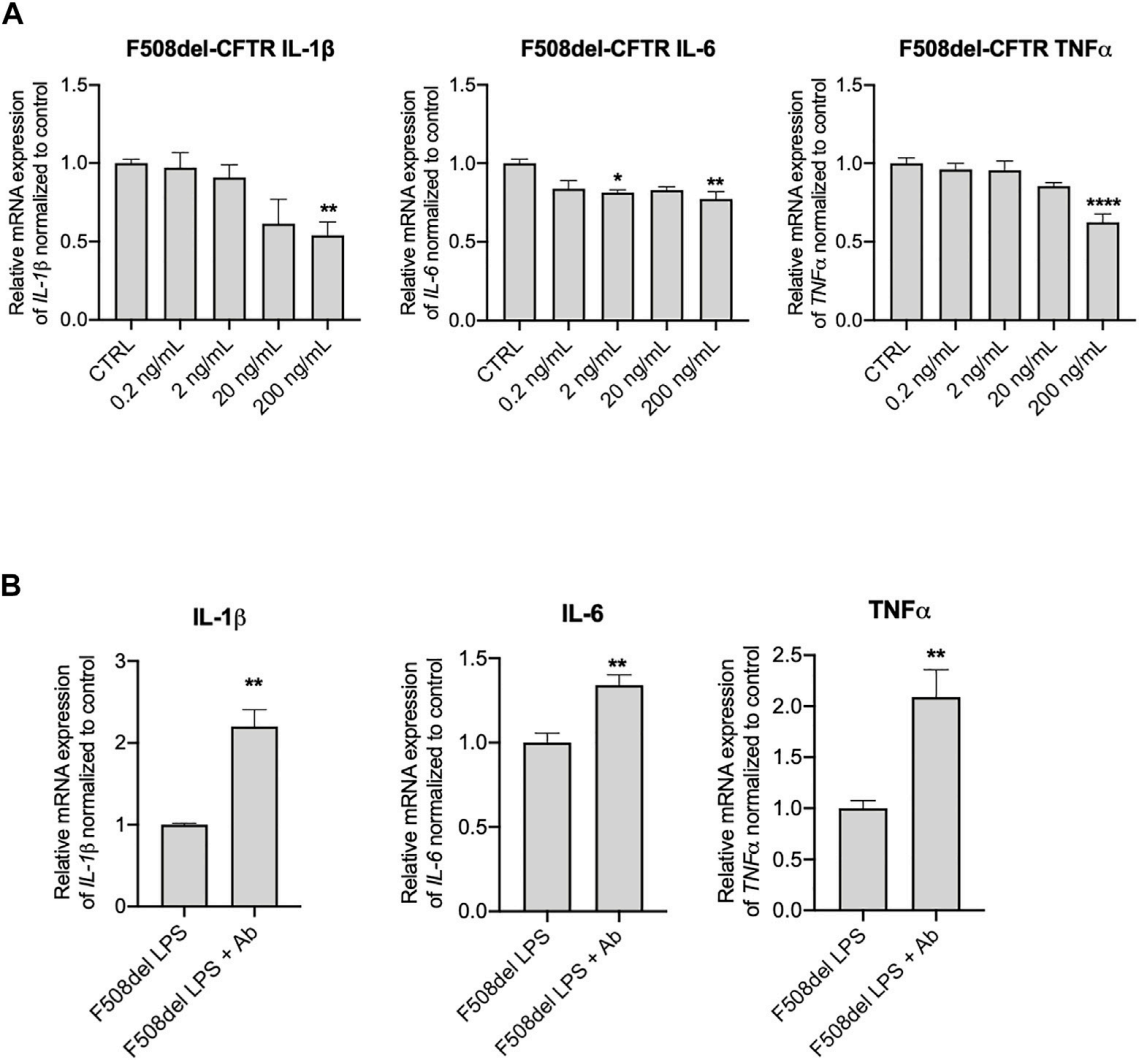
Anti-IGFBP-6 antibodies increased pro-inflammatory cytokines expression. **(A)** F508del-CFTR CFBE cells were treated with PBS or 0.2–200 ng/ml IGFBP-6 for 4 h. Statistical significance tested using two-way ANOVA with Tukey’s multiple comparisons test. **(B)** Cells were treated with 1 μg/ml LPS ± an antibody directed against IGFBP-6 (1 μg/ml) for 4 h. Total RNA was extracted and qRT-PCR was performed in order to quantify IL-1β, IL-6 and TNF-α mRNAs normalized to GADPH as housekeeping gene and then to control untreated cells. Data represent the mean ± SEM (*n* = 3). Statistical significance tested using paired two-tailed *t*-test.

To further investigate the effect of IGFBP-6 in the CF context, we treated the F508del-CFTR CFBE cells with IGFBP-6 (from 0.2 to 200 ng/ml) for 24 h and we then measured the mRNA IGFBP-6 level by qRT-PCR. Despite IGFBP-6 reduced the pro-inflammatory cytokines IL-1β, IL-6 and TNF-α mRNA levels in a dose-dependent manner, IGFBP-6 significantly downregulated the pro-inflammatory cytokines only at the highest concentration (200 ng/ml) (Figure 3A and Supplementary Figure S2A). Moreover, pre-incubation of F508del-CFTR cell with an anti-IGFBP-6 antibody to abolish its anti-inflammatory activity, increased pro-inflammatory cytokines expression under infection with LPS (Figure 3B and Supplementary Figure S2A).

We then investigated the effect of IGFBP-6 on CFTR using the FLIPR membrane potential dye assay to measure CFTR activity in CFBE cell line (Jiang et al., 2021). As shown in Supplementary Figures S2A,B, IGFBP-6 did not affect the Wt-CFTR or F508del-CFTR (data not shown) function in CFBE cells. We also tested the effect of IGFBP-6 on Trikafta™ in F508del-CFTR CFBE cells. The pre-treatment with VX-661+VX-445 resulted in a significant improvement in VX-770 potentiated channel activity (Supplementary Figures S2C,D). Moreover, IGFBP-6 pre-treatment did not affect the Trikafta™-mediated F508del-CFTR function. Immunoblotting analysis confirmed that IGFBP-6 did not modify the Wt- and F508del-CFTR protein expression (Supplementary Figures S2E,F).

### IGFBP-6 Downregulates Pro-inflammatory Cytokine Expression Also in Primary Nasal Epithelial Cells

**FIGURE 4:**
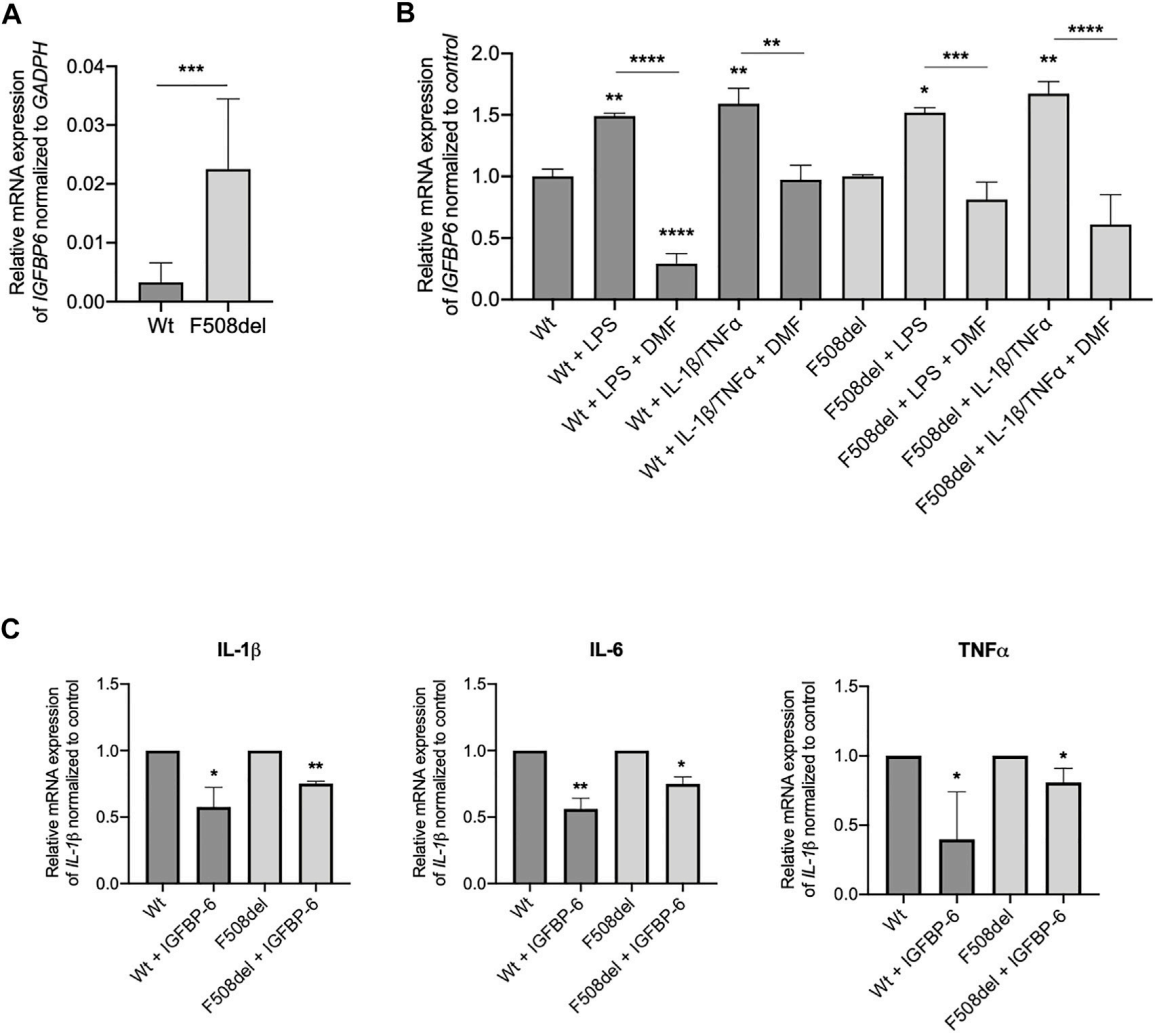
IGFBP-6 shows higher expression in *F508del*/*F508del* nasal epithelial cells from three CF donors and reduces pro-inflammatory cytokines expression. **(A)** Total RNA was extracted from 2 patients homozygous for *F508del* mutation and 2 non-CF donors. qRT-PCR was performed in order to quantify IGFBP-6 mRNA normalized to GADPH as housekeeping gene. Data represent the mean ± SEM (*n* = 4). Statistical significance tested using paired two-tailed *t*-test **(B)** Cells were treated with 0.1% DMSO, 1 μg/ml LPS, 30 ng/ml IL-1β + 30 ng/ml TNFα ± 50 µM DMF for 4 h. Total RNA was extracted and qRT-PCR was performed in order to quantify IGFBP mRNA normalized to control untreated cells. Data represent the mean ± SEM (*n* = 4). **(C)** Cells were treated with 200 ng/ml IGFBP-6 for 4 h. Total RNA was extracted and qRT-PCR was performed in order to quantify IL-1β, IL-6 and TNFα mRNAs normalized to control untreated cells. Data represent the mean ± SEM (*n* = 4). *p < 0.05; **p < 0.01; ***p < 0.001; ****p < 0.0001. Statistical significance tested using two-way ANOVA with Tukey’s multiple comparisons test to the control for (Wt or F508del) each cell line.

Our next goal was to determine whether the IGFBP-6 expression and role observed in bronchial epithelial cell lines translates to patient-derived tissue. We generated epithelial cultures from nasal brushings obtained from 2 non-CF donors and 2 CF patients bearing the *F508del* mutation and differentiated at an air/liquid interface as previously described (Hynds et al., 2019; Laselva et al., 2021c). Interestingly, at basal level, the mRNA IGFBP-6 expression in *F508del/F508del* cells is higher than in non-CF nasal epithelial cells (Figure 4A). Treatment with LPS and IL-1β/TNFα showed a statistically significant increase in mRNA IGFBP-6 expression in both CF and non-CF nasal epithelial cultures and was reduced by DMF co-treatment (Figure 4B).

We next studied the effect of IGFBP-6 on pro-inflammatory cytokines in CF and non-CF nasal epithelial cells. As shown in Figure 4C, IGFBP-6 significantly downregulated the pro-inflammatory cytokines IL-1β, IL-6 and TNFα mRNA levels.

## Discussion

The CF airway inflammatory microenvironment is complex and various cellular and molecular components are involved. The pathogenesis of the inflammatory response in CF can be derived either from the opportunistic bacterial infections or from intrinsic alterations of the inflammatory cells, following lack or dysfunction of CFTR (Cantin et al., 2015). Whatever the origin of the CF inflammatory response, there is no doubt that the chronic damage to the airways is mediated by the massive influx of neutrophils, which are dysregulated in proteases and reactive oxygen species (ROS) production, leading to the respiratory damage. Among the most potent neutrophil chemotactic factors produced in the airway secretions of CF patients, IL-8 (also known as CXCL8) represent one of the central mechanisms in the pathophysiology of the CF pulmonary disease. CF neutrophils, when attracted and activated by IL-8, show various functional alterations, including heightened ROS production, granule exocytosis, retarded apoptosis and deficient intracellular bacterial killing (Guan et al., 2016). All of these alterations contribute to the vicious cycle of deranged inflammation and bacterial infections in the CF airways. However, the system is highly redundant and the appraisal of novel mediators may lead to the discovery of new drug targets or repurposing of old drugs.

Our studies employing two different cell models of the F508del-CFTR mutated protein, began to reveal the role of IGFBP-6 in the CF-associated inflammation in CFBE cell lines and in patient-derived, nasal epithelial cultures. In particular, the basal mRNA and protein IGFBP-6 overexpression in F508del-CFTR as compared with Wt-CFTR cells strongly indicate an intrinsic dysregulation of IGFBP-6 in CF cells, even though we cannot say whether the lack/dysfunction of CFTR may play a role. Other studies, i.e., by downregulating CFTR expression in non-CF cells, might indicate if the IGFBP-6 upregulation is CFTR-dependent. We also found that IGFBP-6 mRNA expression is induced by bacterial-derived stimuli as well as by the pro-inflammatory cytokines after 4h, indicating IGFBP-6 as an early inflammatory mediator. Given the relevance of airway epithelial cells in the initiation and orchestration of the innate immune response of the airways (Whitsett and Alenghat, 2015; Johnston et al., 2021), these findings add a further hint to the possibility to better understand the CF-driven respiratory inflammation.

Besides CFTR modulator-based therapies, no specific anti-inflammatory agents have been developed for the CF lung disease and those available, i.e. corticosteroids and non-steroid ones, are hampered in their efficacy by heavy side effects. The research and development of novel anti-inflammatory agents have been obscured by the discovery of CFTR modulators, thereby inflammation represents a sort of orphan target in CF. DMF, a methyl ester of fumaric acid, is known to reduce cytokine and chemokine gene expression, and to increase anti-inflammatory responses (Stoof et al., 2001; Loewe et al., 2002). These interesting findings have led to increased interest for using DMF in auto-immune or inflammatory diseases, including psoriasis, neurodegenerative diseases and asthma (Seidel and Roth, 2013; Scuderi et al., 2020). We have recently shown that in homozygous F508del CFBE cells, DMF drastically reduced both basal and stimulated expression of the pro-inflammatory cytokines and exerted anti-oxidant effects in the CF cells subjected to oxidative stress by various stimuli (Laselva et al., 2021a). Here, we further explore its anti-inflammatory properties in the context of IGFBP-6, which represents a novel target of DMF. The relevance of IGFBP-6 downregulation by DMF in the context of bacterial and inflammatory-driven IGFBP-6 response is witnessed by the parallel downregulation of IL-8, again further supporting a role for IGFBP-6 in CF inflammation.

Finally, the challenge of bronchial epithelial cell lines with IGFBP-6 decreased the expression of TNFα and IL-1β, suggesting an autocrine role in the attenuation of inflammation when IGFBP-6 is secreted upon bacterial/inflammatory stimuli.

Complex interactions occurring among pro- and anti-inflammatory cytokines in CF airways should be also studied in case of IGFBP-6 before certainly assessing its regulatory role in CF inflammation.

In conclusion, IGFBP-6 represents a novel mediator in CF airway inflammation, and our data suggest a negative feedback autoregulator of the inflammatory response of airway epithelial cells.

## Data Availability

The original contributions presented in the study are included in the article/Supplementary Material, further inquiries can be directed to the corresponding authors.
