# Supplementary figures and images for: Insulin-Like Growth Factor Binding Protein (IGFBP-6) as a Novel Regulator of Inflammatory Response in Cystic Fibrosis Airway Cells
Source: Front Mol Biosci. 2022 Jul 12;9:905468. doi: 10.3389/fmolb.2022.905468 (PMC9322660; doi:10.3389/fmolb.2022.905468)

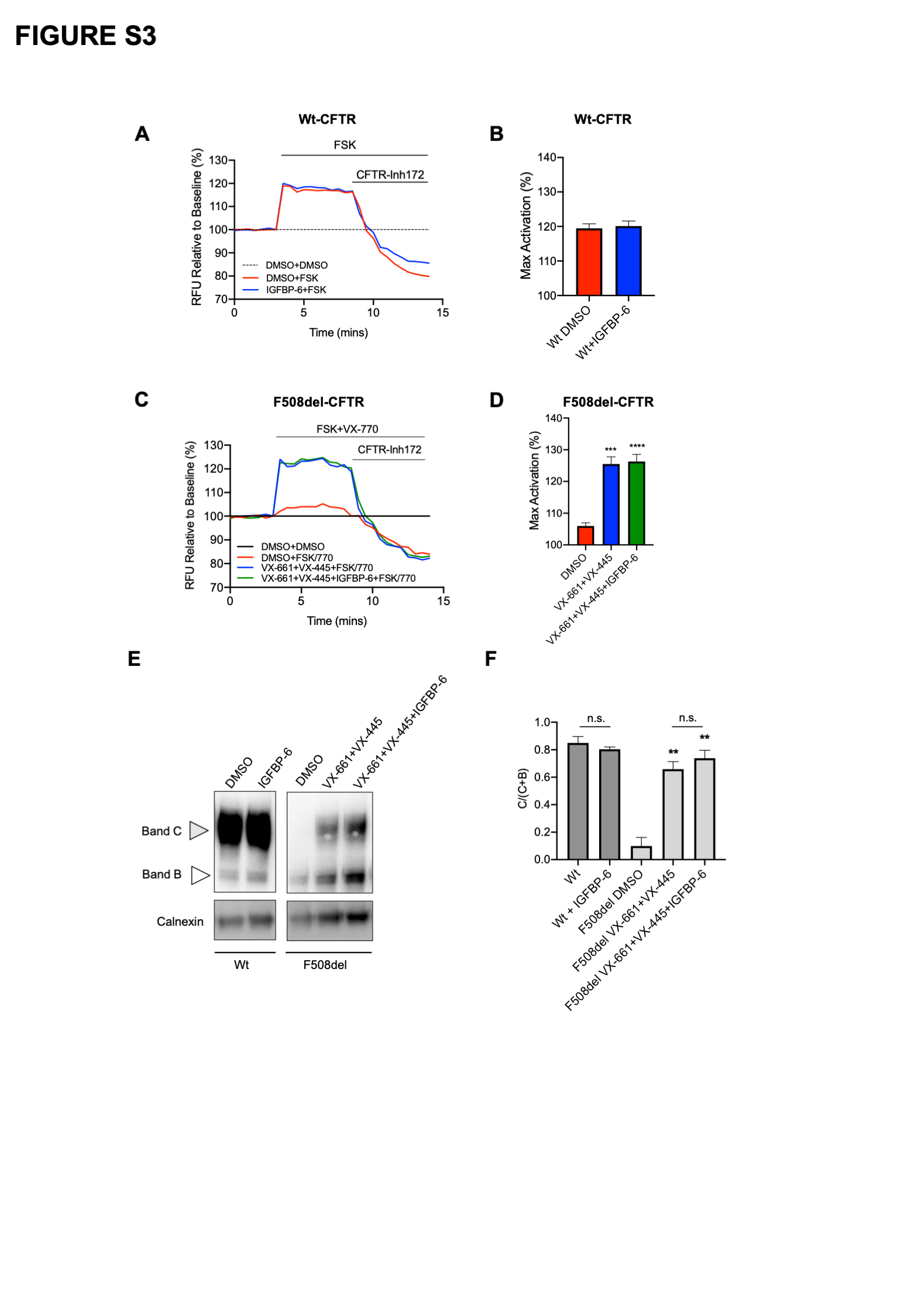

Supplement: Supplementary file 1 [file Image3.tiff]

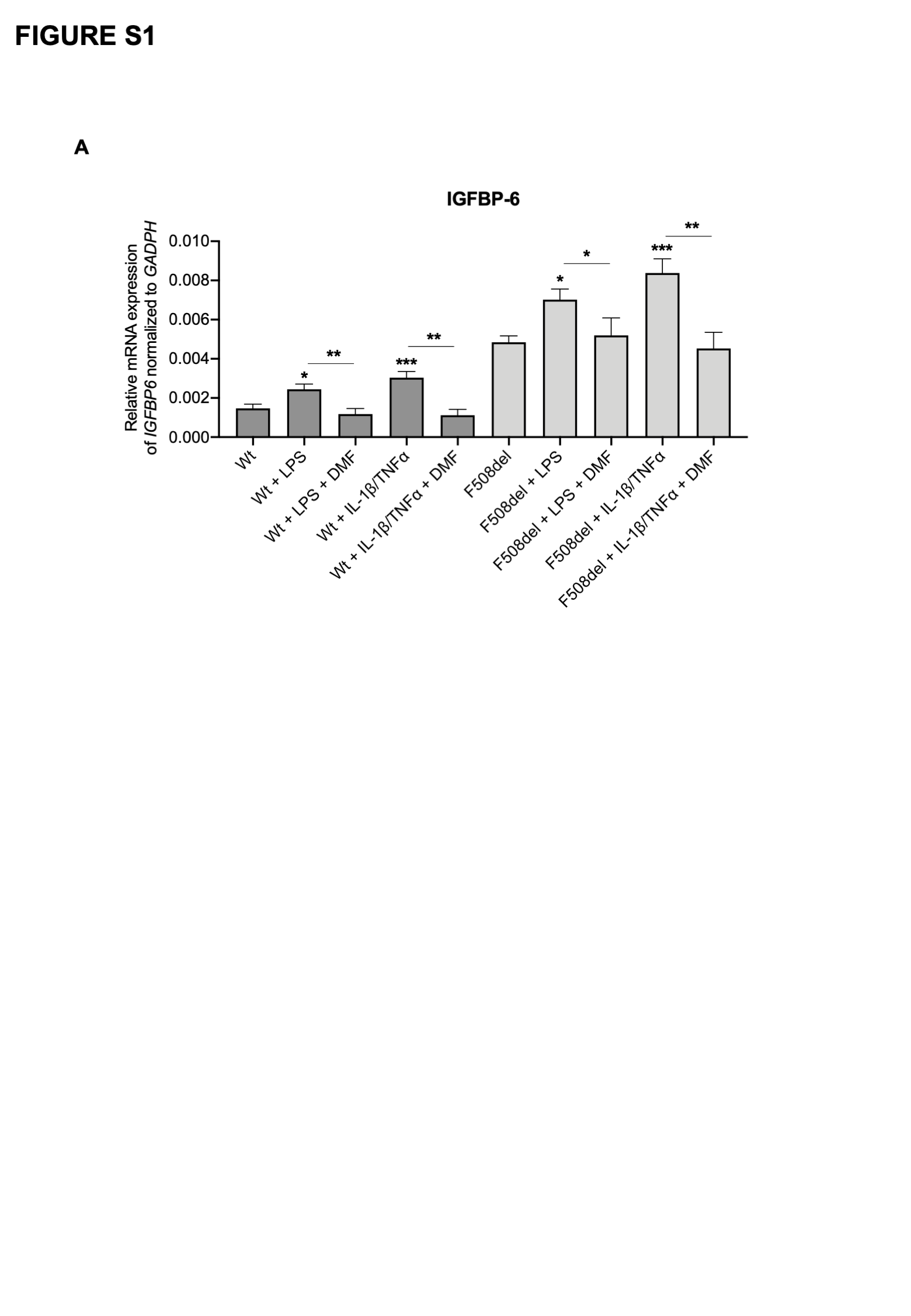

Supplement: Supplementary file 2 [file Image1.tiff]

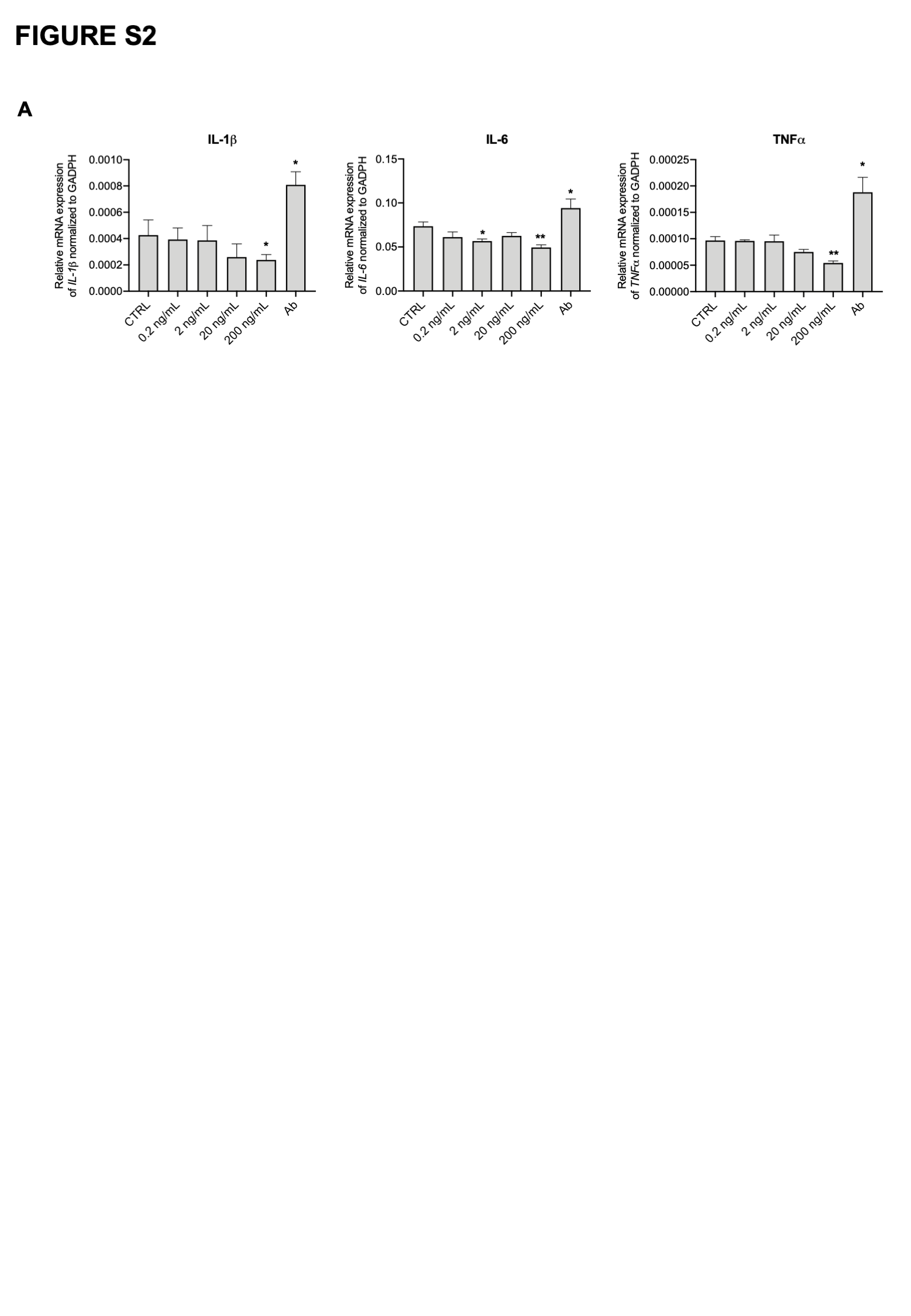

Supplement: Supplementary file 3 [file Image2.tiff]
